# NADP-dehydrogenases contribute to aluminum tolerance through the maintenance of redox homeostasis in soybean

**DOI:** 10.1007/s12298-026-01830-3

**Published:** 2026-10-12

**Authors:** Maria Helena Faustinoni Bruno, Bruno Guilherme Gonçalves, Karla Veloso Gonçalves Ribeiro, Maximiller Dal-Bianco, Laércio Junio da Silva, Wagner Luiz Araújo, Cleberson Ribeiro

**Affiliations:** 1 Departamento de Biologia Vegetal, Universidade Federal de Viçosa, Viçosa, Minas Gerais Brazil; 2 Núcleo de Microscopia E Microanálise, Universidade Federal de Viçosa, Viçosa, Minas Gerais Brazil; 3 Departamento de Bioquímica E Biologia Molecular, Universidade Federal de Viçosa, Viçosa, Minas Gerais Brazil; 4 Departamento de Agronomia, Universidade Federal de Viçosa, Viçosa, Minas Gerais Brazil; 5 Laboratório de Morfofisiologia Vegetal, Departamento de Biologia Geral, Universidade Federal de Viçosa, Viçosa, Minas Gerais Brazil

**Keywords:** Antioxidant system, Ascorbate–glutathione cycle, Enzymatic activity, Gene expression, Oxidative stress, Redox status

## Abstract

Soybean is one of the most important crops globally, however its cultivation in acidic soils is limited by aluminum (Al) toxicity, which impairs root growth and triggers oxidative stress. Maintaining cellular redox balance is essential for Al tolerance to this stress, with NADPH acting as the primary reducing agent in antioxidant systems, such as the ascorbate–glutathione (AsA-GSH) cycle. This study evaluated the role of NADP-dehydrogenase enzymes (G6PDH, 6PGDH, ICDH, and NADP-ME) in response to Al in two contrasting soybean genotypes: NEO610 (Al-tolerant) and M5917I (Al-sensitive). Upon Al exposure, NEO610 exhibited a greater increase in the activity of these enzymes, accompanied by gene expression upregulation particularly for 6PGDH and NADP-ME, elevating NADPH levels and the NADPH/NADP⁺ ratio. The elevated NADPH concentration favored increased activity of AsA-GSH cycle enzymes (APX, MDHAR, DHAR, and GR), promoting efficient regeneration of ascorbate and glutathione levels, maintenance of low H_2_O_2_ levels, and reduction of lipid peroxidation. In contrast, M5917I showed stronger transcriptional responses, particularly for NADP-ME, but lower increases in enzyme activities, which were associated with greater H_2_O_2_ accumulation and oxidative damage under Al exposure. These results suggest that Al tolerance relies on the ability to maintain redox homeostasis through NADP-dehydrogenases, which are essential for sustaining the antioxidant defense system and root growth. Thus, redox metabolism may be associated with aluminum tolerance and could represent a potential target for further investigation in soybean breeding programs aimed at acidic soils.

## Introduction

Soybean (*Glycine max* L. Merrill) is one of the most important crops worldwide, primarily serving as a significant source of protein and oil for both human and animal consumption (Li et al. 2023). The global distribution of this oilseed often exposes its cultivation to acidic soils, where aluminum (Al) toxicity represents a major limiting factor for productivity (Rasheed et al. 2023).

Al-induced stress, in addition to impairing root development, can disrupt cellular and redox homeostasis, resulting in increased synthesis and accumulation of reactive oxygen species (ROS) (Rasheed et al. 2023; Kocjan et al. 2025). To scavenge these ROS, plants have evolved enzymatic and non-enzymatic antioxidant defense systems; however, these mechanisms require reducing power as a substrate, such as nicotinamide adenine dinucleotide phosphate (NADPH) (Zhao et al. 2020).

The Pentose Phosphate Pathway (PPP) is essential for NADPH production through the activity of its NADP-dehydrogenase enzymes, providing reducing power, metabolic intermediates, and biosynthetic precursors (Jiang et al. 2022; Li et al. 2023) to maintain the redox balance of plant cells (Corpas and Barroso 2014; Zhao et al. 2020; Abulfaraj and Baz 2025). The two dehydrogenase enzymes of the PPP, glucose−6-phosphate dehydrogenase (G6PDH, EC 1.1.1.49) and 6-phosphogluconate dehydrogenase (6PGDH, EC 1.1.1.44), utilize NADP⁺ to generate NADPH during the conversion of glucose−6-phosphate to a pentose. This catalysis is directly involved in controlling and maintaining the NADP⁺/NADPH ratio. Beyond their role in plant development, these enzymes are modulated in response to environmental changes. Studies have shown that G6PDH plays a crucial role in responses to abiotic stresses, such as water deficit (Wang et al. 2025), salinity (Wang et al. 2008), cold (Li et al. 2023), and toxic metals (Huang et al. 2025). For instance, alfalfa plants exposed to Al exhibited increased 6PGDH activity, providing intermediates such as sugars and amino acids to the tissues (Fan et al. 2011).

Isocitrate dehydrogenase (ICDH; EC 1.1.1.42) plays a critical role in the tricarboxylic acid (TCA) cycle by catalyzing the oxidative decarboxylation of isocitrate to alpha-ketoglutarate, while also serving as a major source of NADPH (Liu et al. 2010; Mhamdi et al. 2010; Niazi et al. 2019). The NADPH generated by ICDH provides essential reducing equivalents for the regeneration of substrates for thiol-reducing enzymes, such as glutathione (Niazi et al. 2019). Recent studies demonstrate that increased ICDH activity conferred enhanced tolerance to drought in maize (Wei et al. 2023) and to heavy metals, such as cadmium and lead, in *Dianthus spiculifolius* (Liu et al. 2025).

Another notable dehydrogenase is the malic enzyme (NADP-ME, EC 1.1.1.40), whose activity is fundamental in providing NADPH for the biosynthesis of defense metabolites, such as lignin and flavonoids. Furthermore, NADP-ME plays a crucial role in ROS metabolism, mediating responses to both abiotic and biotic stresses (Zhou et al. 2012). Increased NADP-ME activity has been associated with enhanced plant performance under various stressors, including pathogen attack (Voll et al. 2012), salinity (Kandoi and Tripathy 2023), oxidative stress induced by hydrogen peroxide (H_2_O_2_), osmotic stress from mannitol (Zhou et al. 2012), and water deficit (Babayev et al. 2014).

The availability of NADPH is critical for the efficiency of the ascorbate–glutathione (AsA-GSH) cycle and, consequently, for H_2_O_2_ scavenging (Leterrier et al. 2012; Wang et al. 2016). Two key enzymes of this cycle, glutathione reductase (GR) and monodehydroascorbate reductase (MDHAR), utilize NADPH to regenerate glutathione (GSH) and ascorbate (AsA), respectively, which are subsequently employed to scavenge cellular H_2_O_2_ (Liu et al. 2013). Under Al stress, wheat root tips exhibited increased activity and transcript levels of ascorbate peroxidase (APX), GR, and dehydroascorbate reductase (DHAR), coupled with elevated AsA and GSH pools, which contributed to cultivar tolerance (Sun et al. 2014).

The upregulation of NADPH-dehydrogenases and its correlation with the AsA-GSH cycle has been shown to confer greater tolerance in wheat (Slaski et al. 1996), maize (Liu et al. 2010), and *Arabidopsis* (Zhao et al. 2020) by mitigating oxidative damage. However, studies exploring the role of these enzymes in Al stress mitigation in soybean remain scarce (Huang et al. 2025). Therefore, this study investigated the activity and expression of NADPH-producing dehydrogenases in relation to Al stress tolerance in soybean roots. We hypothesize that Al tolerance in soybean relies on the induction of NADP-dehydrogenase activity, which enhances NADPH production and sustains cellular redox homeostasis, thereby improving the efficiency of the AsA-GSH cycle and mitigating oxidative damage.

## Material and methods

### Plant material and cultivation conditions

Seeds of soybean (*Glycine max* L. Merrill) genotypes NEO610 and M5917I were obtained from Neogen and Monsoy, respectively. Seeds were surface-disinfected in a 5% (v/v) sodium hypochlorite solution for 2 min and washed thoroughly with distilled water. The seeds were then placed in Germitest® paper rolls moistened with 250 mL of distilled water (control) or an AlCl₃ solution, with both treatments had their pH adjusted to 4.0, corresponding to nominal Al concentrations of 0 and 1.5 mM, respectively, and kept for a period of 5 days. The pH of the solutions was adjusted using a pH meter by adding 0.1 N HCl or 0.1 N NaOH as necessary. Free Al^3^⁺ activity and Al speciation were not calculated in this study. The plant material was grown in a Mangelsdorf-type germinator with a 16/8 h (light/dark) photoperiod, a light intensity of 30 μmol photons m^−2^ s^−1^, a temperature of 25 ± 2 °C, and 90% relative humidity. On the fifth day, upon reaching the cotyledonary stage, phenotypic evaluations were performed, and roots were collected in liquid N_2_, stored in an ultra-freezer (− 80 °C), and subsequently used for biochemical and molecular analyses.

The use of a 1.5 mM Al concentration and the choice of the two soybean genotypes used in this study were established during a five-day germination period used to screen 65 soybean genotypes (unpublished data). In this preliminary study, several parameters related to seed germination, seedling vigor, and growth under aluminum stress conditions were evaluated. Following the assessment of these parameters, genotypes with contrasting responses to aluminum were selected. Among them, the NEO610 genotype exhibited superior performance compared to M5917I, particularly regarding root length, total seedling length, vigor index, and root surface area. Consequently, these two genotypes were chosen as contrasting models to investigate the physiological and biochemical mechanisms associated with aluminum tolerance.

### Root length and aluminum concentration

The length of the longest root (cm) was measured using a graduated ruler. Subsequently, roots were collected, washed in deionized water, and dried in a forced-air oven at 70 °C until a constant weight was reached, followed by grinding in a knife mill. To measure the Al concentration, 0.2 g of dry root mass was digested in HNO_3_–HClO_4_ (4:1, v/v) at 200 °C, and the concentration was measured using an inductively coupled plasma optical emission spectrometer (ICP-OES, Optima 8300 DV, Perkin Elmer, Waltham, USA).

### RNA extraction, cDNA synthesis, and gene expression by qRT–PCR

Samples of 100 mg of fresh roots were ground in liquid N_2_, and 750 µL of Trizol® LS Reagent (Invitrogen, Thermo Fisher Scientific) was added for RNA extraction. Subsequently, RNA samples were treated with RNase-free DNase I (Invitrogen, Thermo Fisher Scientific). For cDNA synthesis, RNA treated with RNase-free DNase I was used, and oligo(dT)_20_, dNTPs (Invitrogen, Thermo Fisher Scientific), DTT (Invitrogen, Thermo Fisher Scientific), RNaseOUT (Invitrogen, Thermo Fisher Scientific), and M-MLV Reverse Transcriptase (Invitrogen, Thermo Fisher Scientific) were added. Quantitative real-time PCR (qPCR) was performed using iTaq Universal SYBR® Green Supermix (Bio-Rad), cDNA, and primers designed for real-time PCR.

The selected genes and primer sets used are listed in Table 1. Primers were designed using Primer Express Software Version 3.0 and aligned via the SoyBase platform (https://www.soybase.org/). The internal control gene used was *GmActin* (Glyma.18G214200) (FW 5’- TGGACTCTGGTGATGGTGTG-3’ and RV 5’-CTCAGCAGAGGTGGTGAACA-3’). Amplification efficiency for each primer set, including *GmActin*, was verified using a standard curve of serial dilutions, with efficiencies ranging between 98.6% and 102.3%. Additionally, the expression stability of *GmActin* was verified in our experimental samples across all treatments, exhibiting a stable threshold cycle, thereby confirming its suitability for accurate normalization in this study.

**Table 1:** Genes and primers sequences

Gene name	SoyBase identifier	Forward primer (5' *→ * 3')	Reverse primer (5' *→ * 3')
*Gm*G6PDH	Glyma.19G077300	AATGAATCCCCTCTATCG	ACCTCATCTGGTAGTAGG
*Gm*6PGDH	Glyma.19G038400	CAAAACCTTGCTCTCAACATTGC	CGCGGTCCGGTTGTAGAC
*Gm*NADP-ME	Glyma.06G087800	GACAAAAGTTGTTGGCAGTCAT	ACCACCGTTCCTCATCTCCTT
*Gm*ICDH	Glyma.04G211000	GCCCTGATGGAAAGACTATTGAA	CCCTAAAATGGCGGGTAACTG

*GmActin* was used as the internal reference gene for normalization because previous validation studies identified Actin as one of the most stable reference genes in soybean roots subjected to aluminum toxicity (Gao et al. 2017). This choice is further supported by the evaluation of reference genes in soybean root tissues by Wan et al. (2017), who identified *ACT11* and *ELF1B* as highly stable reference genes under abiotic stress conditions.

Amplification reactions were performed in an Applied Biosystems Real-Time PCR System according to the following thermal profile: 5 min at 95 °C, followed by 40 cycles of 30 s at 95 °C, 30 s at 58 °C, and 30 s at 72 °C, and a final extension of 10 min at 72 °C. A melting curve analysis was performed at the end to confirm amplification specificity. Relative expression was calculated using 2^⁻ΔΔCt^ method, according to Livak and Schmittgen (2001).

### Extraction of NADP-dehydrogenases

Root samples (0.1 g) were homogenized in 1 mL of extraction medium consisting of 50 mM HEPES-Tris buffer (pH 7.8), 3 mM MgCl_2_, 1 mM EDTA, and 1 mM PMSF (Wang et al. 2017). Subsequently, the extraction medium was centrifuged at 12,000 g at 4 °C for 20 min, and the supernatant was collected to determine the activities of glucose−6-phosphate dehydrogenase (G6PDH, EC 1.1.1.49), 6-phosphogluconate dehydrogenase (6PGDH, EC 1.1.1.44), isocitrate dehydrogenase (ICDH, EC 1.1.1.42), and NADP-malic enzyme (NADP-ME, EC 1.1.1.40).

### Activity of NADP-dehydrogenase enzymes

To determine G6PDH and 6PGDH activities, 10 μL of the enzyme extract was added to the reaction medium consisting of 50 mM HEPES-Tris buffer (pH 7.8), 3.3 mM MgCl_2_, and 0.5 mM NADPNa_2_. Subsequently, 0.5 mM D-glucose−6-phosphate was added as a substrate for G6PDH, and 5 mM 6-phosphogluconic acid was added for 6PGDH (Wang et al. 2017).

For ICDH determination, the reaction medium consisted of 50 mM HEPES-Tris buffer (pH 7.8), 2 mM MgCl_2_, 0.8 mM NADPNa_2_, and 10 mM DL-isocitric acid (Leterrier et al. 2012). For NADP-ME, the medium consisted of 50 mM HEPES-Tris buffer (pH 7.8), 2 mM MgCl_2_, 0.8 mM NADPNa_2_, and 1 mM disodium L-malate (Muñoz-Vargas et al. 2020).

Enzyme activities were measured at 25 °C by monitoring the reduction of NADP^+^ to NADPH at 340 nm. Specific enzyme activity was expressed in μmol min⁻^1^ mg⁻^1^ protein. Total proteins were determined in the crude extract using BSA as a standard (Bradford 1976).

### NADP and NADPH content

NADP and NADPH contents were quantified according to Zhang et al. (2020). For NADP extraction, 0.1 M HClO₄ was used, followed by centrifugation at 14,000 g for 10 min at 4 °C. The supernatant was neutralized with 0.1 M KOH in 0.2 M Tris–HCl buffer (pH 8.4). For NADPH quantification, the extraction medium consisted of 0.1 M KOH, and the supernatant was neutralized with 0.1 M HClO₄ in 0.2 M Tris–HCl buffer (pH 8.4).

The reaction medium consisted of 0.3 M Tricine/KOH buffer (pH 9.0), 12 mM EDTA, 9 mM glucose−6-phosphate, 0.45 units/reaction of glucose−6-phosphate dehydrogenase, 0.3 mM phenazine ethosulfate, and thiazolyl blue tetrazolium bromide (MTT). Absorbance was determined at 570 nm at 30 °C. NADP and NADPH contents were estimated based on calibration curves prepared with authentic NADP and NADPH standards.

#### Activity of enzymes involved in ascorbate and glutathione metabolism

Root samples (50 mg) were ground in liquid N_2_ and homogenized in the following media: 0.1 M potassium phosphate buffer (pH 6.8), 0.1 mM EDTA, 1 mM PMSF, and 1% (w/v) PVPP for ascorbate peroxidase (APX—EC 1.11.1.11) (Ribeiro et al. 2012); 0.1 M Tris–HCl buffer (pH 7.5), 1 mM EDTA, and 10 mM MgCl_2_ (Nagalakshmi and Prasad 2001) for glutathione peroxidase (GPX—EC 1.11.1.9); 0.1 M potassium phosphate buffer (pH 7.5), 1 mM EDTA, 2 mM DTT, 1 mM PMSF, and 1% PVPP for glutathione reductase (GR—EC 1.6.4.2) (Carlberg and Mannervik 1985); 50 mM potassium phosphate buffer (pH 7.8) and 1 mM ascorbate for monodehydroascorbate reductase (MDHAR—EC 1.6.5.4) and 50 mM Tris–HCl buffer (pH 7.4), 100 mM NaCl, 2 mM EDTA, and 1 mM MgCl_2_ for dehydroascorbate reductase (DHAR—EC 1.8.5.1) (Murshed et al. 2008).

Homogenates were centrifuged at 12,000 g for 15 min at 4 °C, and supernatants were collected. Enzyme activities were determined by adding crude enzyme extract to the respective reaction media: 50 mM potassium phosphate buffer (pH 6.0), 0.8 mM ascorbate, and 1 mM H_2_O_2_ for APX; 50 mM potassium phosphate buffer (pH 7.0), 1 mM EDTA, 0.1 mM NaCl, 1 mM reduced glutathione (GSH), 0.2 mM NADPH, 0.25 mM H_2_O_2_, and 1 unit of glutathione reductase for GPX; 0.1 M potassium phosphate buffer (pH 7.5), 1 mM EDTA, 1 mM oxidized glutathione (GSSG), and 0.1 mM NADPH for GR; 50 mM Tris–HCl buffer (pH 7.6), 0.1 mM NADH, 2.5 mM ascorbate, and 0.4 units of ascorbate oxidase for MDHAR; and 50 mM potassium phosphate buffer (pH 7.0), 2 mM dehydroascorbate, and 5 mM reduced glutathione (GSH) for DHAR.

Enzyme activities were determined by monitoring the change in absorbance during the first minute of reaction at 30 °C. Activity was calculated using the following molar extinction coefficients: APX (290 nm, ε = 2.8 mM^−1^ cm^−1^); GPX (340 nm, ε = 9.6 mM^−1^ cm^−1^); GR (340 nm, ε = 6.22 mM^−1^ cm^−1^); MDHAR (340 nm, ε = 6.2 mM^−1^ cm^−1^); and DHAR (265 nm, ε = 14 mM^−1^ cm^−1^). Protein content was determined by the Bradford (1976) method using BSA as a standard.

#### Determination of ascorbate and glutathione contents

To determine ascorbate (AsA) and dehydroascorbate (DHA) contents, 50 mg of roots were homogenized in 6% (w/v) trichloroacetic acid (TCA) and centrifuged at 15,000 g for 5 min at 4 °C (Kampfenkel et al. 1995). Total ascorbate (AsA + DHA) was determined in extract aliquots supplemented with 0.5 mM DTT and 0.02 M potassium phosphate buffer (pH 7.4), followed by incubation at 42 °C for 15 min. Subsequently, 0.025% (w/v) N-ethylmaleimide, 2.5% (w/v) TCA, 8.4% (v/v) H_3_PO_4_, 0.8% (w/v) 2,2’-dipyridyl, and 0.3% (w/v) FeCl_3_ were added, followed by further incubation at 42 °C for 40 min. After stopping the reaction on ice, absorbance was measured at 525 nm. AsA content was determined as described above, but omitting DTT and N-ethylmaleimide. DHA content was calculated as the difference between total ascorbate and the reduced form (AsA). Contents were determined based on a calibration curve using ascorbate standards.

Total glutathione (GSH + GSSG) was determined according to Griffith (1980). Roots (50 mg) were homogenized in 0.1 M HCl and 1 mM EDTA, then centrifuged at 12,000 g for 10 min at 4 °C. To determine total glutathione, 10 μL aliquots of the extract were added to a reaction medium containing 25 mM sodium phosphate buffer (pH 7.5), 30 μM NADPH, and 0.6 mM DTNB. Samples were then incubated at 30 °C for 5 min with 10 μL of glutathione reductase (50 U mL^−1^), and absorbance was determined at 412 nm. To determine GSSG content, 200 μL of the supernatant was derivatized with 10 μL of 2-vinylpyridine (2-VP) for 60 min at 30 °C. After derivatization, GSSG content was determined as described for total glutathione. GSH content was calculated as the difference between total glutathione and GSSG. Glutathione levels were calculated based on a calibration curve using GSH as a standard.

#### Determination of malondialdehyde (MDA) content

Root samples (50 mg) were homogenized in 1% (w/v) trichloroacetic acid (TCA) and centrifuged at 12,000 g for 15 min at 4 °C. Aliquots of 150 μL of the supernatant were added to a 0.5% (w/v) TBA solution in 20% TCA and incubated in a water bath at 95 °C (Cakmak and Horst 1991). After 30 min, the reaction was stopped, tubes were centrifuged at 10,000 g for 10 min, and the absorbance of the supernatant was measured at 532 and 600 nm. The MDA-TBA complex content was determined using a molar absorptivity coefficient of 155 mM^−1^ cm^−1^ (Heath and Packer 1968).

#### Determination of hydrogen peroxide (H_***2***_O_2_) content

Root samples (50 mg) were ground in liquid N_2_ and homogenized in 0.1% (w/v) TCA. After filtration, the extract was centrifuged at 12,000 g for 15 min at 4 °C. Aliquots of the supernatant were added to a reaction medium containing 100 mM sodium phosphate buffer (pH 7.5) and 1 M potassium iodide, followed by absorbance measurement at 390 nm. The H_2_O_2_ content was determined based on a calibration curve prepared with authentic H_2_O_2_ standards.

#### Experimental design and statistical analysis

Experiments were conducted in a completely randomized design (CRD) with five biological replicates, consisting of two treatments: (1) absence of Al (control) and (2) presence of Al (1.5 mM). The experimental unit consisted of 20 seeds per roll, totaling 200 seedlings per genotype. For the biochemical analyses, five independent biological samples were evaluated. For the molecular analyses, three independent biological samples were used, with three technical replicates for each sample.

Residual normality and homoscedasticity of variances were verified using Shapiro–Wilk and Bartlett tests, respectively, at a 5% significance level (*p* > 0.05). Data were subjected to a two-way analysis of variance (ANOVA), with genotype and aluminum treatment as factors. When significant differences were detected, treatment means were compared via the Scott-Knott test (*p* < 0.05) using SASM-AGRI software (Canteri et al. 2001). Graphical representations were generated using GraphPad Prism version 8.0.0 (GraphPad Software, Inc., San Diego, CA, USA).

## Results

**Fig. 1:**
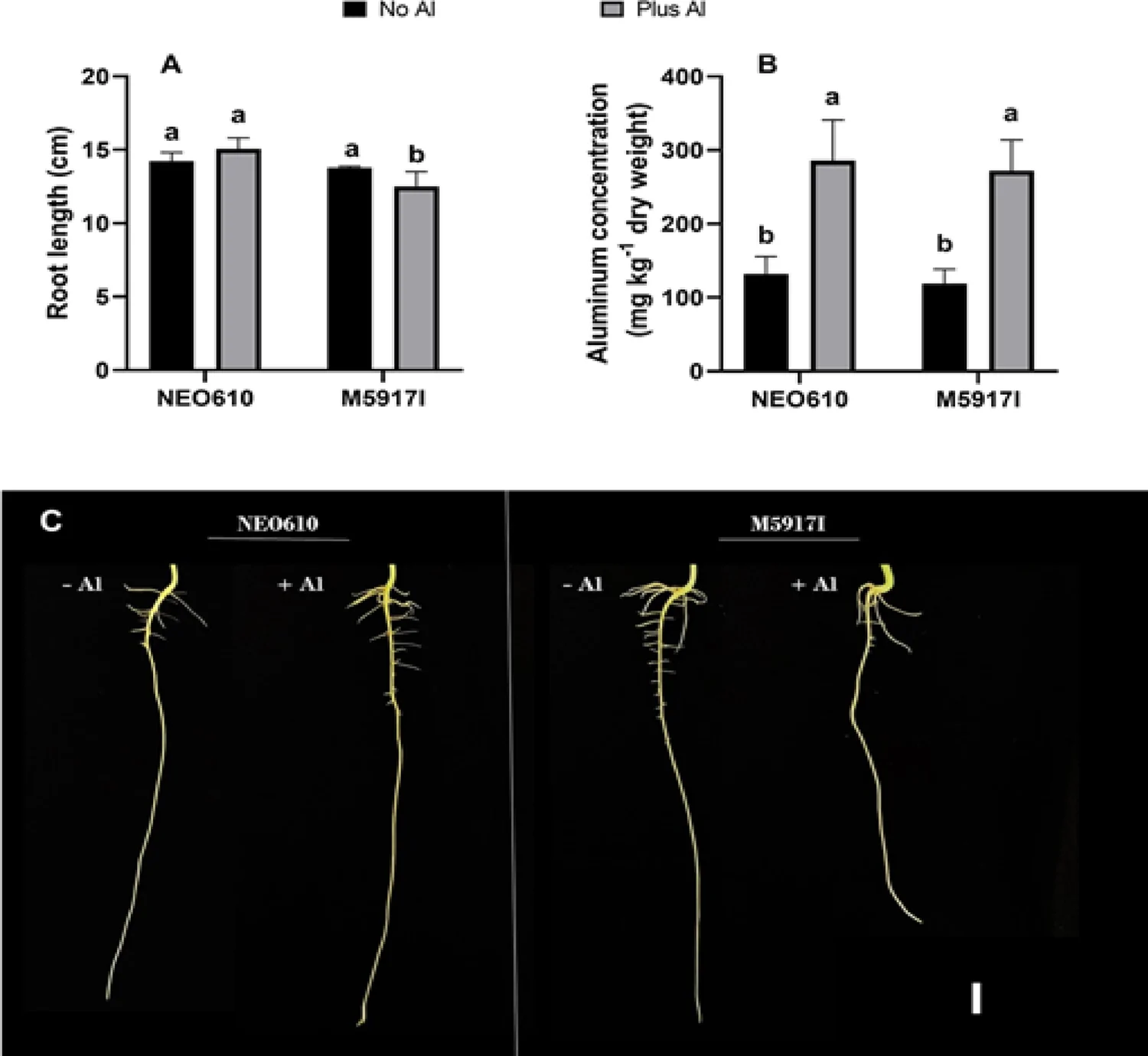
Root length **A**, Al concentration **B** and visual aspect of root system **C** of soybean genotypes NEO610 and M5917I germinated in control treatment 0 mM Al (No Al) and 1.5 mM AlCl_3_ (Plus Al) for 5 days. Different lowercase letters indicate significant differences among all genotype × treatment combinations according to Scott-Knott's test (P < 0.05). Therefore, different letters also indicate significant differences between genotypes within the same treatment. Bars represent standard error. Bar: 1 cm

Root length in the NEO610 genotype was not significantly affected by Al treatment, whereas M5917I exhibited a 14.5% reduction in root length. Thus, Al stress accentuated the contrasting growth responses between the genotypes, with NEO610 maintaining 25.4% greater root growth than M5917I (Fig. 1A, C). Despite these contrasting growth responses, Al treatment increased the metal concentration by 116% and 127% in the roots of NEO610 and M5917I, respectively, indicating a similar capacity for total Al accumulation in the root system (Fig. 1B).

**Fig. 2:**
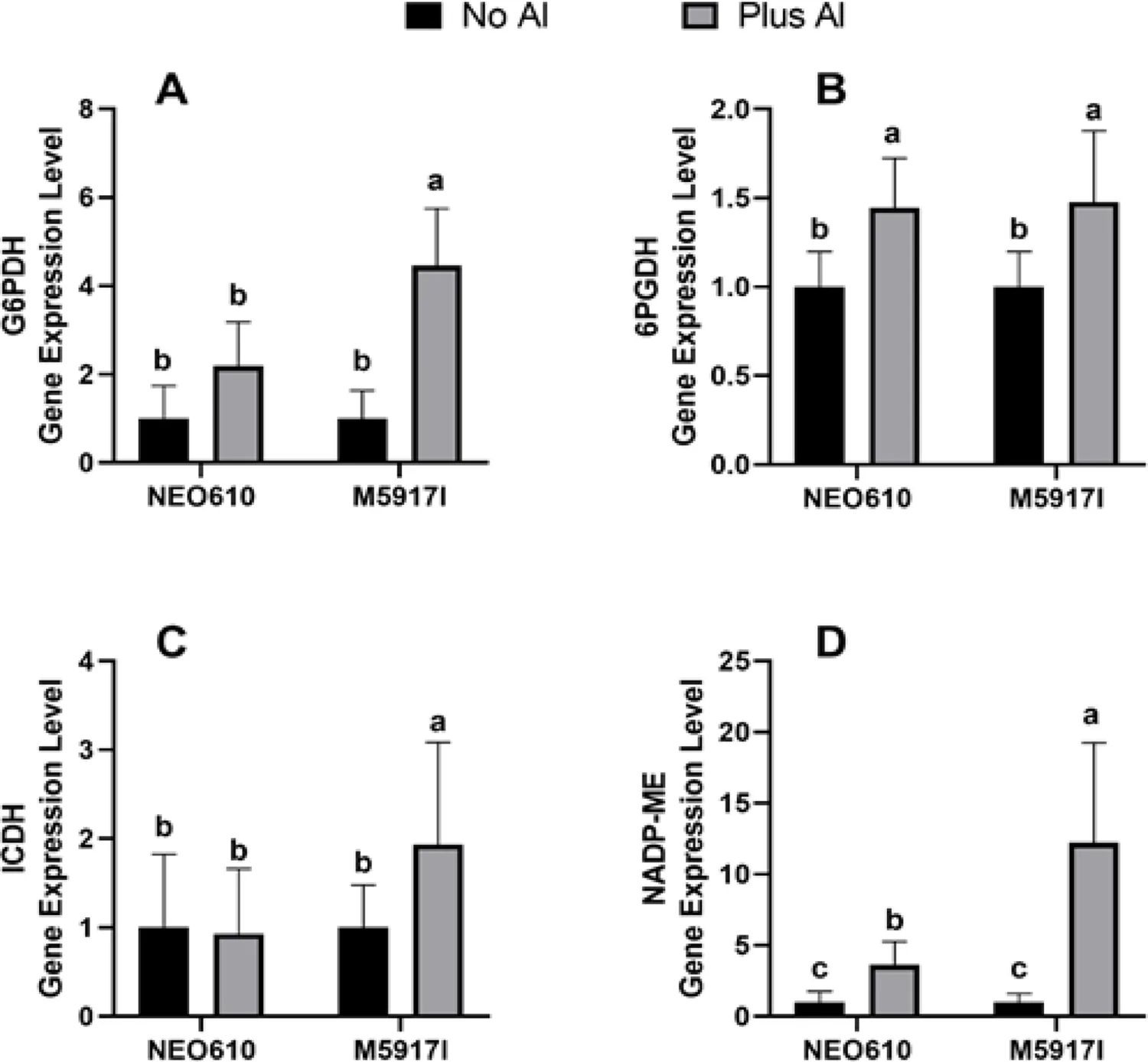
Gene expression NADP-dehydrogenases enzymes in roots of two soybean genotypes NEO610 and M5917I germinated in control treatment 0 mM Al (No Al) and 1.5 mM AlCl_3_ (Plus Al). **A** Glucose−6-phosphate dehydrogenase (G6PDH), **B** 6-phosphogluconate dehydrogenase (6PGDH), **C** Isocitrate dehydrogenase (ICDH) and **D** NADP-ME. Different lowercase letters indicate significant differences among all genotype × treatment combinations according to Scott-Knott's test (P < 0.05). Therefore, different letters also indicate significant differences between genotypes within the same treatment. Bars represent standard error

Al exposure induced distinct transcriptional responses between the two genotypes. *GmG6PDH* and *GmICDH* transcripts were upregulated exclusively in M5917I (Fig. 2A, C), whereas *Gm*6*PGDH* expression increased in both genotypes (Fig. 2B). While *GmNADP-ME* expression was induced in both genotypes, its induction was significantly stronger in M5917I, suggesting genotype-dependent regulation of genes associated with NADPH production (Fig. 2D).

**Fig. 3:**
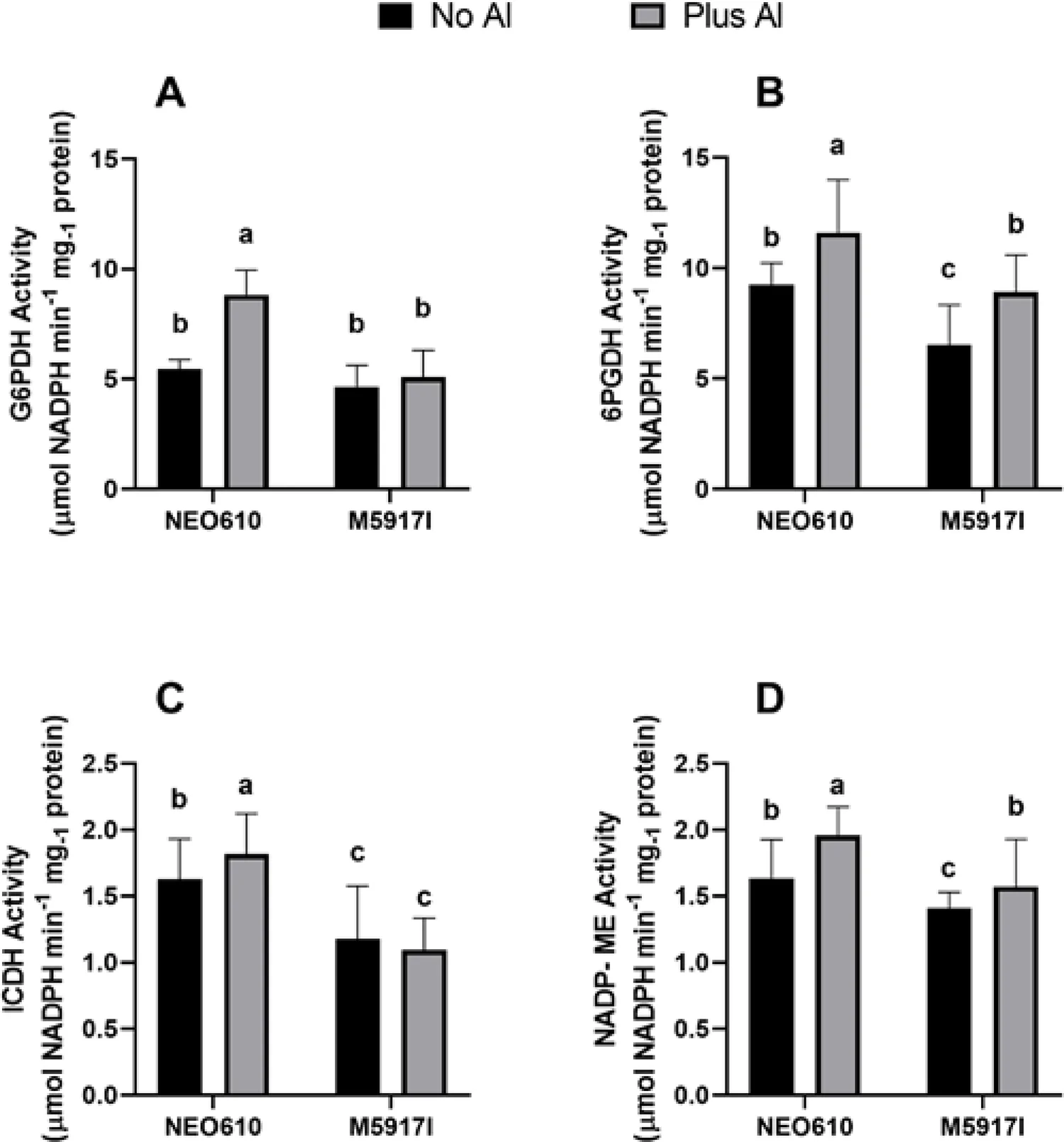
Activity of NADP-dehydrogenases enzymes in roots of two soybean genotypes NEO610 and M5917I germinated in control treatment 0 mM Al (No Al) and 1.5 mM AlCl_3_ (Plus Al). **A** Glucose−6-phosphate dehydrogenase (G6PDH). **B** 6-phosphogluconate dehydrogenase (6PGDH). **C** Isocitrate dehydrogenase (ICDH) and **D** NADP- ME. Different lowercase letters indicate significant differences among all genotype × treatment combinations according to Scott-Knott's test (P < 0.05). Therefore, different letters also indicate significant differences between genotypes within the same treatment. Bars represent standard error

The transcriptional and enzymatic responses showed distinct patterns under Al exposure. Al treatment triggered a generalized increase in the activities of G6PDH, 6PGDH, ICDH, and NADP-ME in NEO610 roots, leading to the highest enzymatic levels observed across both genotypes (Fig. 3). In contrast, the M5917I genotype showed a more restricted response, with activity increments limited to 6PGDH and NADP-ME (Fig. 3B, D). In general, NEO610 exhibited higher constitutive activities of 6PGDH, ICDH and NADP-ME than M5917I even under control conditions, indicating intrinsic metabolic differences between the genotypes (Fig. 3B, C, D).

**Fig. 4:**
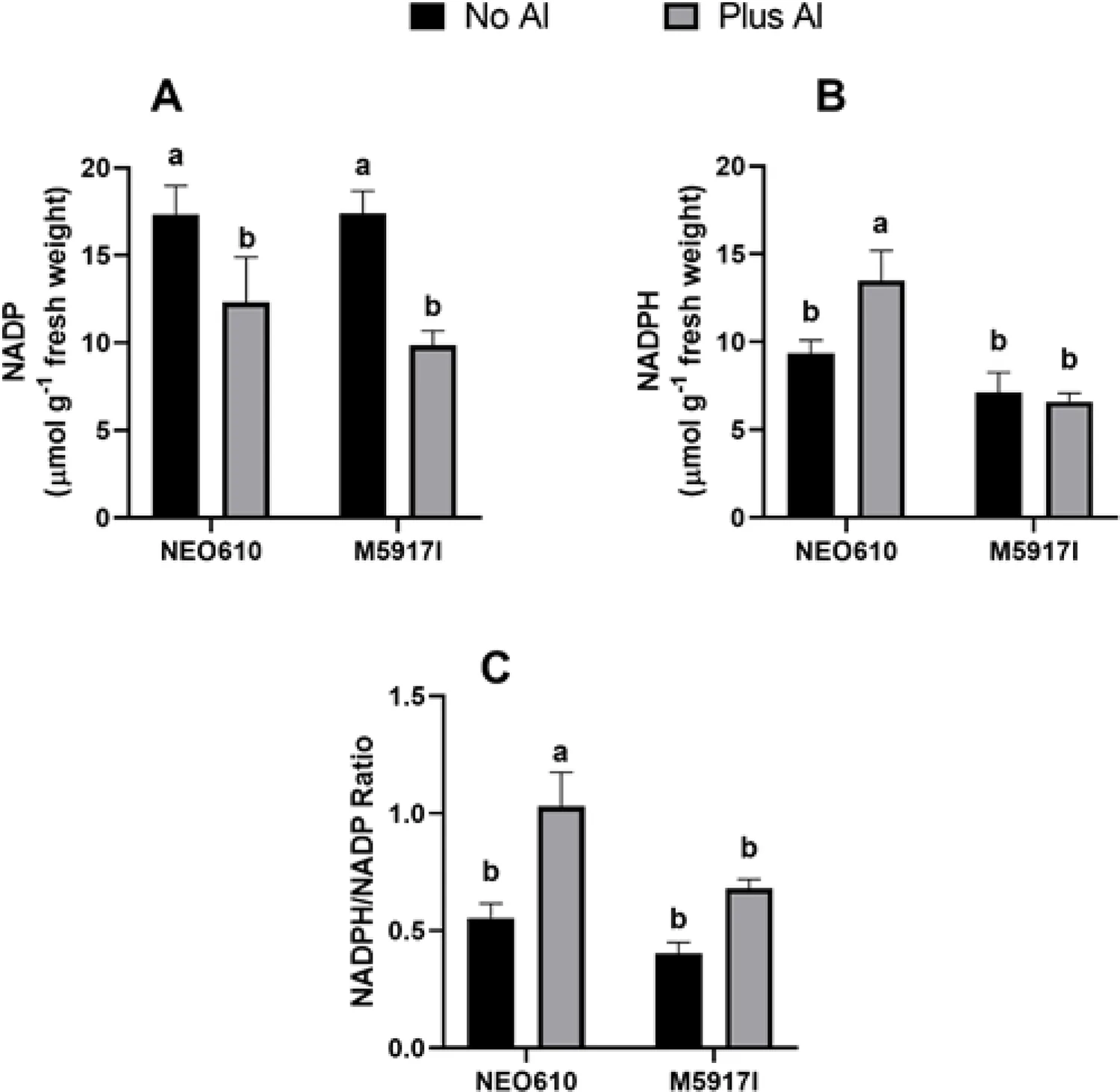
**A** NADP content. **B** NADPH content. **C** NADPH/NADP ratio in roots of two soybean genotypes NEO610 and M5917I germinated in control treatment 0 mM Al (No Al) and 1.5 mM AlCl_3_ (Plus Al). Different lowercase letters indicate significant differences among all genotype × treatment combinations according to Scott-Knott's test (P < 0.05). Therefore, different letters also indicate significant differences between genotypes within the same treatment. Bars represent standard error

Al exposure led to a depletion of the NADP^+^ content in both genotypes (Fig. 4A). However, only NEO610 converted this response into a significant increase in NADPH content, resulting in a marked elevation of the NADPH/NADP^+^ ratio (Fig. 4B, C). In contrast, the absence of NADPH accumulation in M5917I limited changes in its redox ratio, which remained significantly lower than that observed in NEO610 under Al stress (Fig. 4C).

**Fig. 5:**
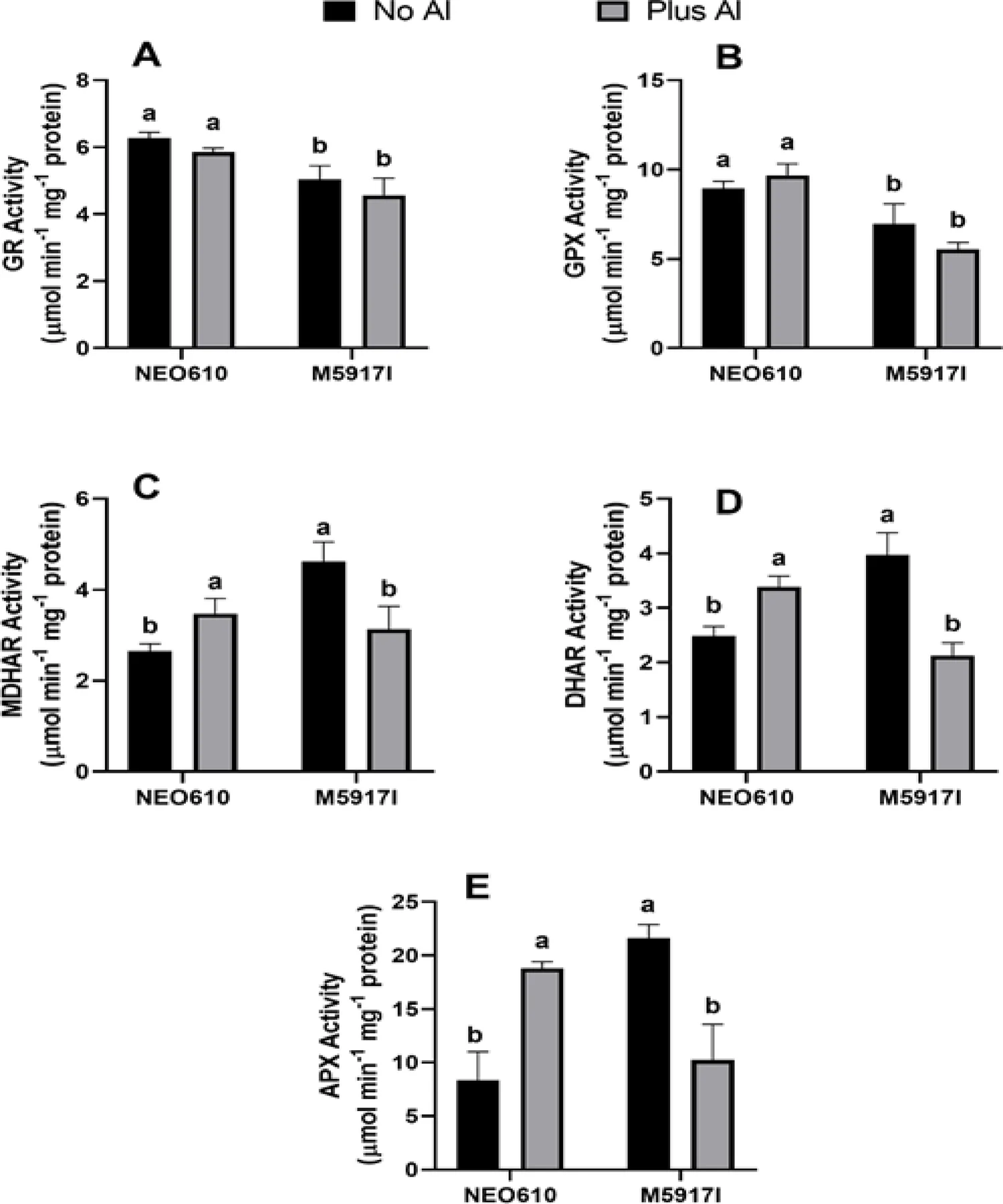
Enzyme’s activity of antioxidant system in roots of two soybean genotypes NEO610 and M5917I germinated in control treatment 0 mM Al (No Al) and 1.5 mM AlCl_3_ (Plus Al). **A** Glutathione Reductase (GR), **B** Glutathione Peroxidase (GPX), **C** Monodehydroascorbate Reductase (MDHAR), **D** Dehydroascorbate Reductase (DHAR) and **E** Ascorbate Peroxidase (APX). Different lowercase letters indicate significant differences among all genotype × treatment combinations according to Scott-Knott's test (P < 0.05). Therefore, different letters also indicate significant differences between genotypes within the same treatment. Bars represent standard error

The activity of glutathione metabolism enzymes, specifically GR and GPX, remained unaffected by Al treatment; although both enzymes consistently exhibited higher activity in NEO610 than in M5917I (Fig. 5A, B). In contrast, the ascorbate recycling pathway responded specifically in the tolerant genotype, with MDHAR, DHAR, and APX activities increased exclusively in NEO610 roots following Al exposure, whereas a decline was observed in M5917I (Fig. 5C, D, E). Among these enzymes, APX showed the greatest difference between genotypes, reaching activity levels approximately two-fold higher in NEO610 under Al stress (Fig. 5E).

**Fig. 6:**
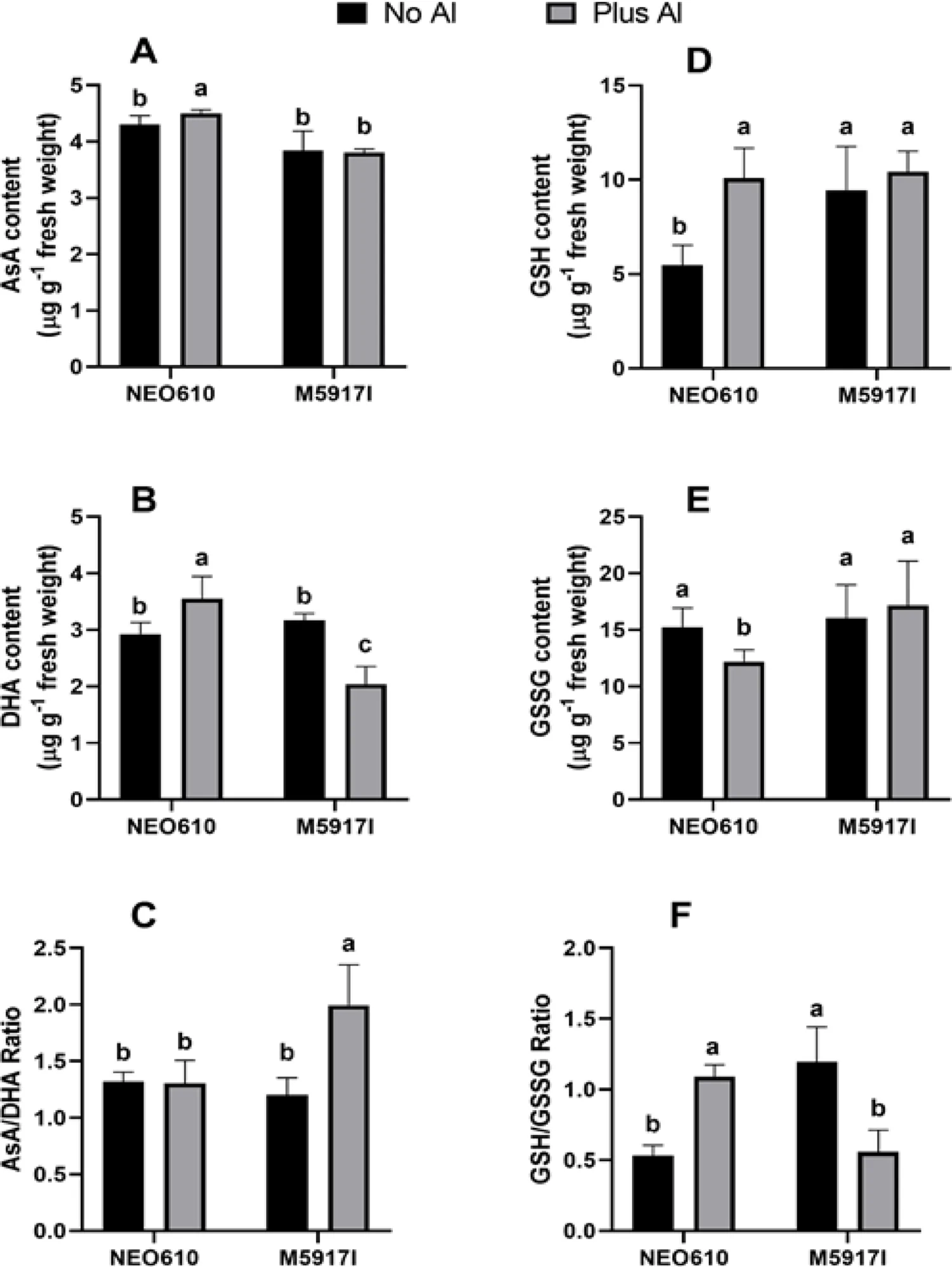
Ascorbate and glutathione concentration in roots of two soybean genotypes NEO610 and M5917I germinated in control treatment 0 mM Al (No Al) and 1.5 mM AlCl_3_ (Plus Al). **A** Ascorbate (AsA), **B** Dehydroascorbate (DHA), **C** AsA/DHA ratio, **D** Reduced Glutathione (GSH), **E** Oxidized Glutathione (GSSG) and **F** GSH/GSSG ratio. Different lowercase letters indicate significant differences among all genotype × treatment combinations according to Scott-Knott's test (P < 0.05). Therefore, different letters also indicate significant differences between genotypes within the same treatment. Bars represent standard error

Al exposure altered the redox metabolism of ascorbate and glutathione in a genotype-dependent manner. AsA levels increased only in the roots of the NEO610 genotype (Fig. 6A). Regarding DHA content, Al treatment induced opposing responses between genotypes, increasing in NEO610 while decreasing in M5917I, resulting in DHA levels 43% higher in the tolerant genotype under Al stress (Fig. 6B). The AsA/DHA ratio increased exclusively in M5917I roots under Al stress, a trend primarily driven by the observed reduction in DHA concentration (Fig. 6C).

GSH concentration rose by 48% exclusively in NEO610 roots following Al treatment (Fig. 6D). In contrast, Al exposure reduced GSSG levels by 20% only in the tolerant genotype (Fig. 6E). These changes were reflected in a higher GSH/GSSG ratio in NEO610, whereas M5917I maintained the lowest glutathione redox status under Al exposure (Fig. 6F).

**Fig. 7:**
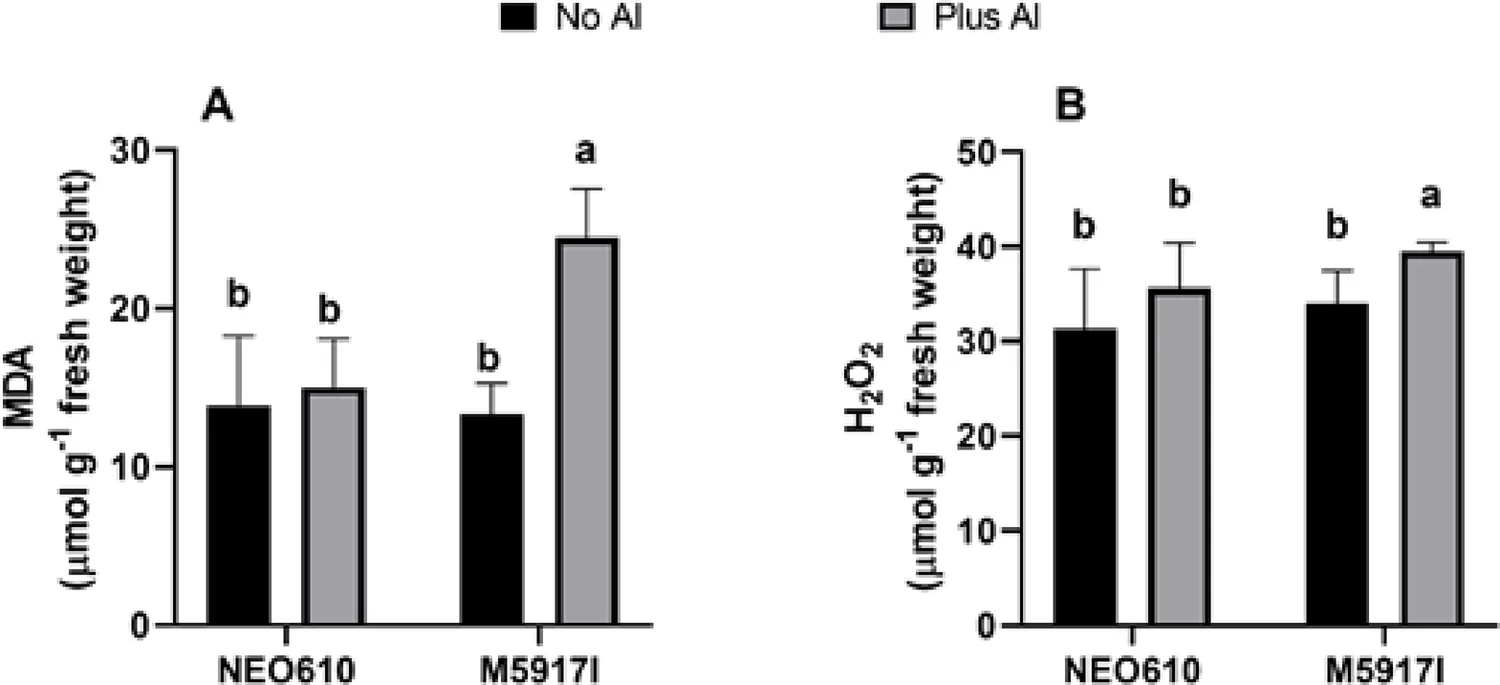
**A** Malonaldehyde content (MDA). **B** Hydrogen peroxide content (H_2_O_2_) in roots of two soybean genotypes NEO610 and M5917I germinated in control treatment 0 mM Al (No Al) and 1.5 mM AlCl_3_ (Plus Al). Different lowercase letters indicate significant differences among all genotype × treatment combinations according to Scott-Knott's test (P < 0.05). Therefore, different letters also indicate significant differences between genotypes within the same treatment. Bars represent standard error

These contrasting antioxidant responses were accompanied by differences in H₂O₂ and MDA levels between the genotypes. MDA concentration increased only in the roots of the M5917I genotype after Al exposure, showing a 30% rise compared to its respective control (Fig. 7A). Under Al stress, a significant accumulation of H_2_O_2_ was observed exclusively in M5917I roots (Fig. 7B). In contrast, the NEO610 maintained its H_2_O_2_ levels similar to those of its control even under Al exposure (Fig. 7B).

## Discussion

Aluminum toxicity primarily impairs root elongation; however, the magnitude of this inhibitory effect is governed by the genotypic capacity to orchestrate robust metabolic defense mechanisms. In the present study, the differential response to Al stress was closely linked to the superior performance of the Al-tolerant genotype (NEO610), characterized by a marked increase in the activity of NADP-dependent dehydrogenases (G6PDH, 6PGDH, ICDH, and NADP-ME), accompanied by the transcriptional upregulation of genes such as *6PGDH* and *NADP-ME*. This metabolic shift led to a significant accumulation of NADPH and an enhanced NADPH/NADP+ ratio, providing the reducing power required to drive the antioxidant defense system, particularly the ascorbate–glutathione cycle. Conversely, the Al-sensitive genotype (M5917I) failed to activate these mechanisms effectively, resulting in exacerbated oxidative damage and impaired root development. These findings underscore that Al tolerance in soybean is intrinsically coupled with the maintenance of redox status, where the supply of reducing equivalents serves as a metabolic bottleneck for sustaining antioxidant pathways under Al toxicity.

Al toxicity primarily targets the root apex, leading to an inhibition of elongation; in this study, the sensitive genotype (M5917I) exhibited a significant reduction in root growth, a response that was absent in the tolerant genotype (NEO610). Root growth inhibition is a hallmark of Al stress in seedlings, particularly in sensitive cultivars (Brito et al. 2020; Ribeiro et al. 2022; Yoshida et al. 2023). Generally, this differential growth response is attributed to a higher Al accumulation in sensitive cultivars compared to tolerant ones (Brito et al. 2020; Ribeiro et al. 2022; Yoshida et al. 2023; Rasheed et al. 2023). However, our results demonstrate that Al concentrations in the roots did not differ significantly between the two genotypes following exposure. These findings suggest that Al tolerance in these soybean genotypes involves the plant’s ability to process and respond to accumulated Al at the molecular and metabolic levels, in addition to potential exclusion mechanisms. However, measuring Al content across the entire root system represents a methodological limitation that may conceal localized differences in Al accumulation within the first few millimeters of the root apex. Consequently, root-apex quantification or spatial imaging techniques would be required to conclusively evaluate Al exclusion mechanisms. However, once accumulated within root cells, Al exposure triggers a rapid disruption of cellular homeostasis, characterized by an exacerbated production of reactive oxygen species (ROS), causing oxidative stress and damage to essential cellular components such as nucleic acids, membrane lipids, and proteins (Liu et al. 2022; Ofoe et al. 2023). Among the diverse response mechanisms, the maintenance of plant redox status through the efficient utilization and maintenance of reducing power stands out as a critical determinant of Al resilience (Chakraborty et al. 2024; Jiang et al. 2025).

The activity of NADPH-generating dehydrogenases was significantly induced by Al exposure, with a more robust and coordinated response observed in the NEO610 genotype. G6PDH, a key enzyme of the oxidative pentose phosphate pathway (OPPP), plays a pivotal role in NADPH production, contributing significantly to plant adaptation under stress conditions (Zhang et al. 2026). The modulation of the OPPP not only sustains essential physiological processes for plant development but also constitutes a first line of defense against diverse environmental stresses (Li et al. 2023). Indeed, enhanced G6PDH activity is a conserved response in plants exposed to stressors such as NaCl, NaHCO_3_, PEG, and mannitol (Yang et al. 2019). In this study, the superior activation of G6PDH in the tolerant genotype aligns with findings in drought-stressed soybean cultivars, where higher G6PDH activity distinguishes tolerant from sensitive genotypes by ensuring the steady supply of reducing equivalents (Liu et al. 2013; Wang et al. 2016).

In the present study, the enhanced G6PDH activity under Al stress suggests its fundamental role in providing reducing equivalents (NADPH) to sustain metabolic defense pathways. Notably, a divergence was observed between *GmG6PDH* transcript levels and total enzymatic activity; while the sensitive genotype showed gene expression induction, this did not translate into higher catalytic capacity. This phenomenon may be attributed to the specificity of the isoform monitored via RT-qPCR (*LOC100806107-Glyma.19G077300*) or suggest differential post-translational regulation between genotypes. In the tolerant genotype, the rapid enzymatic activation independent of de novo transcript synthesis points toward a more efficient metabolic strategy for the immediate maintenance of the NADPH pool.

Although previous reports correlate increased *GmG6PDH* expression with salt stress tolerance (Yang et al. 2019; Zhao et al. 2020), our data indicate that, under Al toxicity, robust catalytic activity is the critical determinant. Huang et al. (2025) reinforce that cytosolic G6PDH alleviates oxidative damage in soybean through NADPH supply, corroborating the enzyme's importance in mitigating ROS accumulation and protecting membranes, as observed in the NEO610 genotype. Furthermore, the coordinated induction of 6PGDH in NEO610 under Al stress underscores the OPPP efficiency. The regulation of this pathway through 6PGDH has been shown to be effective in alfalfa under Al stress (Fan et al. 2011), whereas its overexpression conferred cold tolerance in *Arabidopsis* (Tian et al. 2021). In rice, the link between G6PDH and salinity tolerance was associated with the modulation of response genes (Peng et al. 2024). In soybean, the involvement of the *Gm6PGDH* gene in root system development and the enhancement of the enzymatic antioxidant system was crucial for ROS scavenging (Li et al. 2023), in addition to providing essential intermediates such as sugars and amino acids to sustain growth under stress (Fan et al. 2011).

ICDH represents another vital dehydrogenase source of reducing power which, similar to G6PDH and 6PGDH, showed significant increases in activity in the NEO610 genotype under Al stress. As a component of the Tricarboxylic Acid (TCA) cycle, ICDH possesses chloroplastic, cytosolic, and mitochondrial isoforms, with its cytosolic form being fundamentally involved in providing NADPH for cellular redox status maintenance (Niazi et al. 2019). It has been reported that NADP-ICDH activity increases under salinity-induced oxidative stress (Leterrier et al. 2012) and contributes to sustaining NADPH levels during pathogen attacks in *Arabidopsis* (Mhamdi et al. 2010). Zhang et al. (2026) demonstrated that cytosolic ICDH can regulate plant stem cell homeostasis in response to nutrient deficiency. Furthermore, the overexpression of *ICDH* in *Dianthus spiculifolius* enhanced tolerance to heavy metals, such as cadmium and lead, by increasing NADPH levels and facilitating antioxidant system efficiency (Liu et al. 2025). The role of ICDH also extends to energy metabolism; in rice, increased ICDH activity and expression, along with other TCA cycle enzymes, accelerated metabolic flux and ATP production, providing sufficient energy to sustain germination under water stress (She et al. 2024). Consequently, our results suggest that the increased ICDH activity in NEO610 may contribute to the mechanism involved in Al-induced stress, serving as a crucial source of NADPH for plant redox balance.

*GmG6PDH* and *GmICDH* showed increased expression in M5917I under Al exposure. However, this increase was not accompanied by a corresponding increase in enzyme activity. Although Zhao et al. (2020) reported a positive correlation between *GmG6PDH2* expression and G6PDH activity, the authors also demonstrated that soybean possesses multiple cytosolic and plastidial G6PDH isoforms with distinct expression patterns. Thus, total G6PDH activity may reflect the contribution of different isoforms and may not necessarily reflect the expression of a single isoform, as evaluated in the present study. A similar difference between gene expression and enzyme activity was reported by Long et al. (2016) in *Hevea brasiliensis*, where changes in G6PDH activity showed no clear relationship with the expression of the *HbG6PDH3* and *HbG6PDH4* isoforms following ethylene treatment, suggesting that post-transcriptional and/or post-translational mechanisms may contribute to the regulation of G6PDH activity.

Similarly, Leterrier et al. (2007), when evaluating pea plants subjected to different abiotic stresses, observed that changes in NADP-isocitrate dehydrogenase (NADP-ICDH) expression were not always accompanied by corresponding changes in enzyme activity. The authors suggested that NADP-ICDH may be regulated at different levels, including transcriptional and post-translational mechanisms, depending on the stress condition. In this context, the higher ICDH activity observed in NEO610 together with the higher expression observed in M5917I, may reflect the complex relationship between transcript abundance and enzyme activity during the response to Al stress. These transcriptional responses should, however, be interpreted with caution, considering that a single reference gene was used for normalization in the present study.

NADP-ME activity and expression were enhanced in the roots of both genotypes under Al stress. This enzyme plays a multifaceted role in plant defense, providing reducing power and carbon skeletons for biosynthetic pathways, while assisting in the maintenance of redox balance through the Tricarboxylic Acid (TCA) cycle (Taboada et al. 2023). The importance of NADP-ME in abiotic stress resilience is well-documented; its overexpression in *Arabidopsis* conferred resistance to NaCl, NaHCO_3_, mannitol, and H_2_O_2_ (Zhou et al. 2012), whereas in tobacco (*Nicotiana tabacum*), activity was induced in response to water deficit (Doubnerová-Hýsková et al 2014). Furthermore, the overexpression of *Zea mays* chloroplastic NADP-ME in *Arabidopsis* resulted in reduced malate levels, stomatal conductance, and transpiration rates, favoring salinity tolerance (Kandoi and Tripathy 2023). In the context of metal phytotoxicity, increased activity of this enzyme is strategic for malate homeostasis and root exudation, acting in the chelation of metal ions and mitigating cellular damage (Song et al. 2022). Our results suggest that NADP-ME activation in soybean under Al stress reinforces the NADPH supply, complementing the action of other dehydrogenases in sustaining antioxidant defenses.

It is noteworthy that significant differences between NEO610 and M5917I were also observed under control conditions for some parameters (6PGDH, ICDH and NADP-ME activity), indicating constitutive physiological differences between the genotypes. These basal differences may contribute to their contrasting responses to aluminum stress, as inherent variations in metabolism and growth can influence the capacity to cope with adverse environmental conditions. Therefore, the superior performance of NEO610 under aluminum stress may be associated with both constitutive metabolic traits and its ability to maintain higher enzymatic activities under stress.

The coordinated increase in the activity of NADPH-generating dehydrogenases resulted in a significant rise in NADPH concentration and, consequently, in the NADPH/NADP⁺ ratio, exclusively in the roots of the NEO610 genotype under Al stress. G6PDH and 6PGDH are recognized as the primary sources of NADPH in plant metabolism (Yang et al. 2019). In our study, the enhanced activity of both enzymes in the presence of Al highlights that these dehydrogenases constitute an essential mechanism to ensure the supply of reducing power under stress conditions. In soybean, the overexpression of *GmG6PD7* has been reported to elevate NADPH, AsA, and GSH concentrations in roots exposed to heavy metals, resulting in the mitigation of oxidative damage (Huang et al. 2025). While NADP⁺ content decreased in both genotypes under stress, the NADPH/NADP⁺ ratio increased only in NEO610, reaching values near 1.0. This thermodynamic balance demonstrates that the tolerant genotype successfully maintained the supply of reducing equivalents required to sustain its metabolic defense pathways. Therefore, Al toxicity modulates the interconversion between oxidized and reduced forms of the cofactor, with the maintenance of high NADPH pools being a distinctive trait of Al-tolerant soybean genotypes.

NADP-dependent dehydrogenases generate reducing power in the form of NADPH, an essential substrate for vital metabolic pathways, including enzymatic and non-enzymatic antioxidant defense systems, such as the AsA-GSH cycle (Corpas and Barroso 2014; Wang et al. 2016; Lu et al. 2025). This cycle constitutes the primary pathway for ROS scavenging in plants, relying on the coordinated activity of four central enzymes (APX, MDHAR, DHAR, and GR) and the antioxidant metabolites ascorbate and glutathione (Bashir et al. 2022; Ansari et al. 2024). In our study, the higher NADPH content in the roots of the NEO610 genotype contributed to the superior efficiency of the AsA-GSH cycle under Al stress. In this genotype, a robust increase in the activities of APX, MDHAR, and DHAR was observed, along with higher GR and GPX activities. Consequently, the regeneration of reduced forms of ascorbate and glutathione was optimized, resulting in increased concentrations of these metabolites in the presence of Al and the maintenance of redox homeostasis.

Within the AsA-GSH cycle, ascorbate metabolism is driven by the sequential activity of three enzymes: APX, MDHAR, and DHAR (Noctor 2025). The enzymes MDHAR and DHAR are crucially involved in the regeneration of ascorbate (AsA), with MDHAR directly requiring the reducing power of NADPH to convert monodehydroascorbate (MDHA) back to AsA (Zhou et al. 2021; Ansari et al. 2024; Singh et al. 2024). Previous reports indicate that enhanced G6PDH activity is vital for supplying NADPH to the AsA-GSH cycle, thereby modulating enzymatic activities and enhancing the overall antioxidant capacity of plants (Wang et al. 2016). Consequently, the increased NADPH concentration—driven by the superior activity of NADP-dependent dehydrogenases in the NEO610 genotype contributed to the efficient activation of this pathway under Al stress, as evidenced by the significant rise in AsA levels.

Additionally, the enzyme DHAR catalyzes the conversion of dehydroascorbate to AsA using GSH as a reducing agent, and its increased activity has been reported as an important Al-tolerance mechanism (Sun et al. 2014). The maintenance of the GSH pool relies crucially on the GR enzyme, which utilizes NADPH as a cofactor to reduce oxidized glutathione (GSSG) (Lu et al. 2025). In barley, G6PDH was shown to favor alkaline stress tolerance, biomass production, and photosynthetic activity by mediating NADPH production and the subsequent accumulation of GSH (He et al. 2021). NADPH is therefore essential for sustaining adequate intracellular GSH levels, a molecule that plays a central role in regulating the homeostasis of reactive oxygen species in plants under stress (Tian et al. 2021). In rice, higher GR activity in Al-tolerant genotypes promoted an elevation in GSH content, requiring a continuous supply of NADPH (Ribeiro et al. 2022). Our results corroborate these observations, indicating that the higher GSH concentrations in NEO610 roots under Al stress derive from the synergy between elevated GR activity and the greater availability of NADPH generated by NADP-dehydrogenases. In this context, the OPPP consolidates itself as a vital source of reducing power, playing a decisive role in adaptive responses to stress (Chaput et al. 2020).

Concomitant with the activity of enzymes involved in GSH and AsA regeneration are the enzymes that consume these metabolites, such as GPX and APX, respectively (Ansari et al. 2024). In the NEO610 genotype, APX activity increased following Al treatment, while GPX maintained its activity at levels significantly higher than those of the sensitive genotype. Both enzymes are involved in the consumption of reactive oxygen species, eliminating H_2_O_2_ from cellular metabolism (Wang et al. 2008; Zhou et al. 2021). The efficient regeneration of AsA and GSH in NEO610 provided a steady supply of substrates for APX and GPX, allowing these enzymes to function with high catalytic capacity. Consequently, this genotype managed to maintain H_2_O_2_ homeostasis and stable lipid peroxidation rates. Conversely, in the Al-sensitive genotype M5917I, the lower activity of APX and GPX resulted in H_2_O_2_ accumulation and increased lipid peroxidation. Thus, our results indicate that a robust ascorbate and glutathione metabolism is pivotal in preventing Al-induced oxidative damage in tolerant soybean cultivars.

In summary, Al phytotoxicity extrapolates mere metal accumulation in tissues and is intrinsically linked to the genotypic capacity to sustain redox homeostasis. Although both genotypes accumulated similar Al levels, only NEO610 demonstrated an efficient adaptive response. In this genotype, the enhanced total catalytic activity of NADP-dehydrogenases ensured a steady supply of NADPH and an elevated NADPH/NADP⁺ ratio, providing the reducing power required for antioxidant pathways, even though the transcriptional upregulation of specific studied isoforms was not always positively correlated with total enzyme activity. Consequently, the Ascorbate–Glutathione cycle operated with high efficiency, preserving AsA and GSH pools, which prevented H_2_O_2_ accumulation and lipid peroxidation. In contrast, the M5917I genotype was unable to sustain this metabolic apparatus, resulting in compromised redox status, severe oxidative damage, and root growth inhibition. While localized Al exclusion at the root apex cannot be entirely ruled out, the absence of differences in whole-root Al concentration suggests that the contrasting responses observed between the soybean genotypes may involve mechanisms beyond Al exclusion. These responses may be associated with an integrated metabolic network in which NADP-dehydrogenases contribute to the maintenance of the antioxidant system. The ability of the NEO610 genotype to maintain a steady NADPH flux under stress suggests that the regulation of these NADPH-generating enzymes is a key genetic component. Finally, the observed discrepancy between the transcript abundance of specific isoforms (such as G6PDH and ICDH, which were upregulated in the sensitive genotype) and the total enzymatic activity suggests a complex regulatory network. While this disconnect hints at potential post-translational modifications or differential isoform contributions, further studies quantifying protein abundance are required to confirm these mechanisms.

Therefore, metabolic engineering or marker-assisted selection focusing on modulating the activity and efficiency of the NADP-dehydrogenase pathway, emerges as a promising strategy. Rather than focusing solely on organic acid exudation, breeding for redox status robustness offers an essential second line of defense, ensuring that root integrity is maintained even in soils with high aluminum saturation.

## Data Availability

All data generated or analyzed during this study are included in this published article.
